# Passive Immunisation in the Treatment of Infectious Diseases Related to Highly Potent Bacterial Toxins

**DOI:** 10.3390/biomedicines12122920

**Published:** 2024-12-23

**Authors:** Marta Prygiel, Ewa Mosiej, Karol Wdowiak, Aleksandra Anna Zasada

**Affiliations:** National Institute of Public Health NIH—National Research Institute, Chocimska 24, 00-791 Warsaw, Poland; emosiej@pzh.gov.pl (E.M.); kwdowiak@pzh.gov.pl (K.W.); azasada@pzh.gov.pl (A.A.Z.)

**Keywords:** passive immunisation, antitoxins, toxins, botulism, tetanus, diphtheriae

## Abstract

The discovery of microbial toxins as the primary factors responsible for disease manifestations and the discovery that these toxins could be neutralised by antitoxins are linked to the birth of immunology. In the late 19th century, the serum or plasma of animals or patients who had recovered from infectious diseases or who had been immunised with a relevant antigen began to be used to treat or prevent infections. Before the advent of widespread vaccination campaigns, antitoxins played a key role in the treatment and prevention of diseases such as diphtheria and tetanus. A significant reduction in mortality following the introduction of antitoxins confirmed their efficacy. Serum therapy remains an important measure for post-exposure prophylaxis and for the treatment of unvaccinated or incompletely vaccinated patients. For the botulinum toxin, antitoxin therapy continues to be the sole available treatment. The manuscript contains a summary of the most important information on the passive immunoprophylaxis used in the treatment of diphtheria, tetanus, and botulism, all representing diseases in which symptoms are driven by the activity of highly potent bacterial toxins.

## 1. Introduction

Passive immunisation is used to prevent and treat diseases associated with immunodeficiency disorders, severe acute infections, and poisonings in non-immune patients. Passive immunity can be acquired either naturally (via the maternal placental route) or artificially (through transfusion). Artificial passive immunisation involves administering high titres of specific antibodies to an infectious agent or toxin which may originate from human, animal, or genetically engineered sources.

Passive immunisation has been used in clinical practice since the late 19th century, when Emil Adolf von Behring pioneered the use of animal antibodies for the treatment of diphtheria. Behring observed that blood from immunised guinea pigs, rabbits, sheep, goats, and horses with inactive or sublethal diphtheria cultures prevented infection in non-immune animals [1]. This method was rapidly adopted in clinical practice, leading to a significant reduction in mortality during diphtheria outbreaks. His groundbreaking work on immunoserum therapy earned von Behring the first Nobel Prize in Medicine in 1901 [2]. Behring made a similar discovery using *Clostridium tetani*. Blood from rabbits immunised with *C. tetani* protected mice against the tetanus toxin or against a lethal dose of virulent tetanus bacteria. These findings laid the foundations for the production of a tetanus antitoxin [1,2]. Initially, antibodies were obtained from immunised animals such as rabbits, mice, guinea pigs, goats, sheep, horses, and even cows [1,2]. From the 1920s to the 1970s, passive immunisation was also used extensively for the treatment of the whooping cough and the scarlet fever [3]. The classic method for producing antibodies involved immunising animals with specific antigens and isolation-specific antibodies from the blood. Unfortunately, animal-derived proteins often induced adverse reactions, necessitating the development of alternative antibody production methods. Despite the advance of modern technologies in relation to the acquisition of specific immunoglobulins, some of them are still derived from animal sources.

The method of fractionating human plasma into proteins to obtain human blood-derived medicinal products was developed by Cohn and his colleagues in the 1940s and remains in use today in a modified form [4]. The first human immunoglobulin introduced in 1944 for the treatment and prevention of measles, for example, continues to be employed. The incidence of serum sickness and related adverse reactions has been significantly reduced [5]. Currently, two types of human immunoglobulin preparations are used: normal and specific immunoglobulins. Human normal immunoglobulin contains the IgG antibodies, typically found in the general population. It is usually prepared from pooled plasma from at least 1000 donors and exhibits a distribution of IgG immunoglobulin subclasses similar to that found in healthy human plasma [6]. This preparation is most commonly used in primary and secondary immunodeficiency syndromes, including AIDS [7]. Specific immunoglobulin is a concentrated formulation of antibodies with a wide range of specificities against various pathogens. It contains at least 90% IgG and is produced by collecting blood from patients recovering from specific diseases [6]. Specific immunoglobulins are employed in the treatment of the CMV virus, hepatitis A and hepatitis B viruses, measles, rabies, the varicella zoster virus, and tetanus [8]. Human immunoglobulins are also used in patients with primary immune thrombocytopenia, the Kawasaki syndrome, and to prevent feto-maternal incompatibility and the development of hemolytic disease of the fetus and newborn [8]. The source of antibodies is freshly frozen plasma collected from donors in hospitals and blood donation centres. Plasma for antibody fractionation undergoes rigorous screening tests for blood-borne viruses, such as hepatitis B, hepatitis C, and the human immunodeficiency viruses, to prevent the transmission of infections [8]. Additionally, since the 1980s, both plasma and medicinal products derived from pooled plasma have been subjected to inactivation procedures to eliminate potential infectious agents [8]. Inactivation methods ensure protection from previously known infectious agents and from those yet unrecognized. Currently, there are three registered methods for blood pathogen inactivation: the Theraflex MB-Plasma system using methylene blue, the Intercept system using amotosalen, and the Mirasol system which is based on riboflavin [9]. All of them are based on a photodynamic inactivation procedure involving the inhibition of pathogen replication. The Mirasol system is the most commonly used [9]. Each batch of human immunoglobulins undergoes quality control testing in an independent control laboratory to guarantee its consistency with the strict requirements, including the exclusion of microbial contaminants (e.g., endotoxins) [10]. Human plasma varies in its content of specific antibodies, with the selection of plasma depending on the therapeutic goals. In human plasma, the quantity of active glycoproteins depends on individual exposure to antigens, but, most often, the mean concentration is about 7–12 g/L [11]. The therapeutic effect of immunoglobulins is dose-dependent [12]. Therefore, depending on the disease, different doses of human immunoglobulins are administered (low or high) [13]. Low doses have a pro-inflammatory effect and are most often used as a replacement therapy in relation to immunodeficiencies. High doses (above 1 g/kg of body weight) have immunomodulatory and anti-inflammatory effects [13]. Immunoglobulins in high doses are used, most often, in association with haematological and autoimmune disorders, as well as rheumatic inflammatory, infectious, and neurological disorders [13]. Specific immunoglobulins are often used as a hyperimmune therapy against specific infectious agents to neutralise specific pathogenic antigens [13]. Therefore, the doses are selected individually for the patient’s benefit and depend on many factors, including the type of disease, route of administration, and patient weight [13]. Medicinal products containing human immunoglobulins, administered intravenously (IVIG), intramuscularly (IMIG), or subcutaneous (SCIG), are widely used. Each route of immunoglobulin administration, i.e., IVIG, IMID, or SCIG, offers different benefits for the individual [14]. IVIG requires fewer infusion sites and less frequent infusions than IMID and SCIG. However, IMID and SCIG do not require venous access and are associated with fewer systemic adverse infusion reactions than IVIG [14]. The advantage of IMID and SCIG therapies is also that they can be self-administered at home. This is very important for improving the quality of life of patients with primary immunodeficiency diseases who often require long-term or even lifelong treatment [14]. Nonetheless, IVIG preparations penetrate the blood faster, so the therapeutic effects are visible much earlier. Therefore, the intramuscular route is often used after exposure, and the intravenous route is often used therapeutically [14].

Plasma collected from individuals with a history of infections (referred to as convalescent plasma, or CP) is also utilised in treatments. For example, CP has been used in the treatment of patients with COVID-19 [8], particularly in relation to patients in the early stages of the infection [15]. It has been shown that high-titre CP can reduce hospitalization rates by up to 54% in outpatients when administered within a short window (preferably within five days of diagnosis). This treatment has been especially helpful for immunocompromised patients or in areas with limited access to alternative therapies. Despite mixed results in larger trials, CP remains an important tool for managing COVID-19 in certain patient groups [15].

With the advancements in monoclonal antibody (mAbs) production technologies, new products for passive immunisation have been developed. Monoclonal antibodies were first discovered in 1975 by George Köhler and Cesar Milstein, who described a system for obtaining pure antibodies of known specificity in large quantities through “hybridoma technology”. For this revolutionary discovery in clinical medicine and biological research, they were awarded the Nobel Prize in Medicine in 1984 [16]. mAbs are immunoglobulins designed with an identical amino acid sequence that binds to a specific epitope on an antigen. The antigen-binding region of the mAb is designed to confer high specificity to the target antigen and is formed by the variable domains of heavy and light chains. The original “hybridoma technology” method involved the hyperimmunisation of mice with the desired antigen and the isolation of the spleen containing the proliferating B cells. To immortalise B cells in in vitro cultures, fusion with non-secretory myeloma cells was performed. The resulting hybridomas were capable of secreting large quantities of the desired antibody into the culture supernatants [17]. However, rodent-derived monoclonal antibodies proved to be less effective than anticipated when used as a treatment. They activated human defense systems poorly and were targeted by the host’s immune response, leading to significantly shortened half-lives in circulation [17]. Creating human monoclonal antibodies at the time posed several challenges. Immortalised human B cells were unstable, tended to lose the ability to produce antibodies, and produced antibodies in low quantities, reducing antigen affinity [17]. Furthermore, the hyperimmunisation of humans was not feasible, and antigenic stimulation of cultured human lymphocytes in vitro proved to be unreliable. Peripheral blood, the most accessible source of human lymphocytes, was found to be a poor source of B lymphocytes producing specific high-affinity antibodies. B lymphocytes are more abundant in the lymph nodes, bone marrow, and spleen. Today, mAbs, initially produced through the “hybridoma technology”, are produced by recombinant DNA technology using expression systems in mammalian cells. It is possible to produce human recombinant mAbs using phage display technologies as well as transgenic animals with human immunoglobulin genes [17]. Many preclinical safety evaluations and the early-phase clinical trials for mAb, including immunogenicity and pharmacokinetic testing, are ongoing [18]. The challenges associated with the development and use of mAbs can lead to potential regulatory non-compliance if not managed properly. These issues may arise at various stages of research, development, and commercialisation. Here are some key concerns: study population misalignment, inadequate dose justification, and pharmacokinetic differences. These challenges can result in significant delays in the drug registration process and its market introduction if not addressed appropriately during the development and research phases [19]. Fortunately, despite the above challenges arising from the selection of the most appropriate study population, initial dose determination, routes of administration, and potential pharmacokinetic differences between healthy individuals and patients expressing the target antigen, many mAbs have, thus far, received marketing authorisation for therapeutic use. mAbs are now used not only to treat infectious diseases, but also for the treatment of arthritis and inflammatory bowel disease and as part of the immunotherapy for many cancer types, including the treatment of solid tumours [18].

Nanobodies (Nbs) are antibody fragments derived from heavy-chain IgG antibodies, each possessing a single variable antigen-binding domain and found in the Camelidae family. Conventional antibodies consist of two heavy chains, each consisting of three constant domains and one variable domain, and two light chains, each consisting of one constant domain and one variable domain [20]. These single-domain variable heavy-chain (VHH) antibodies were discovered for the first time in 1989 by Professor Raymond Hamers Casterman of the Brussel University in dromedary camels infected with *Trypanosoma evansi* [21]. Despite their simple structure, these antibodies have unique properties such as high solubility and remarkable stability, even in extreme conditions (e.g., over a wide range of pH and temperatures) [22]. VHHs retain very high antigen-binding affinity and specificity comparable to that of mAbs or often even higher [23]. Due to their small size, they have the ability to recognise hidden antigenic sites that are inaccessible to conventional antibodies. Their small size also ensures good penetration into the tissue and rapid distribution, both of which can be very useful in treating toxin-dependent diseases when the time of drug administration is a very important prognostic factor [23]. Many of the advantages of Nbs over conventional mAbs and mAb fragments have already found success in diagnostic disease detection tools such as LFIAs, ELISAs, and biosensors [20]. VHHs have also been explored for a variety of therapeutic applications, including the treatment of cancer, autoimmune diseases, neurological disorders, and haematology [24]. The first drug based on VHHs for the treatment of immune thrombotic thrombocytopenic purpura was approved by the US FDA in 2018 [20]. It has also been shown that, when optimised through genetic engineering, VHHs can be used in the treatment of infectious diseases, but their implementation still requires a lot of work, research, and development [20]. It is, therefore, certain that VHHs will play an important role in the next generation of therapeutics in the future. However, clinical applications are still being explored, and further research is required to determine their efficacy and safety in humans.

Recently, recombinant antibodies or antibody fragments (including nanobodies), produced through the phage display library, have emerged as important research tools. Phage display is a technique that involves the expression of specific peptides or proteins (antibodies or single-chain variable fragments (scFvs) of antibodies) on the surface of bacteriophages [25]. Most often, phage libraries are based on scFvs, which are the smallest functional antibody fragments responsible for antigen interactions. Antibody libraries are generated by preparing template cDNA from B cells of non-immunised donors, followed by the amplification of the heavy- and light-chain antibody fragments. A diverse array of antibody variants can be generated on the surface of the phages. Screening these phage libraries enables the selection of the specific phage that interacts with the selected antigen. This technique mimics the natural process of antibody production with an increase in the affinity of the antibodies with the antigen. The use of phages is a fast and cost-effective alternative to mAb production, as antibodies can be produced entirely in vitro, without requiring an immunisation procedure. Additionally, this method allows for the improvement of existing mAbs and utilises human antibody genes as a source for phage libraries. This circumvents the need for complex techniques to humanise animal-derived antibodies [25].

In the following sections of the paper, we present key information on the passive immunoprophylaxis used in the treatment of botulism, tetanus, and diphtheria—diseases characterised by symptoms resulting from the action of highly potent bacterial toxins. Therefore, the purpose of botulism, tetanus, and diphtheria therapies is to inactivate the circulating toxin and prevent its binding to tissues by using a passive immunisation treatment as a post-exposure approach and/or prophylaxis. Currently, the primary treatment for botulism, tetanus, and diphtheria consists of the administration of antitoxins, which can neutralize the toxins. Antibiotics are used very rarely and not in every case of illness. Due to the fact that diphtheria is a disease transmitted by droplets, to limit transmission and hasten the clearance of the bacteria from the organism, penicillin or, alternatively, erythromycin should be administered [26]. Antimicrobial agents are not currently used for the treatment of botulism, due to the potential lysis of bacterial cells and the release of the botulinum toxin (BoNT) into the intestinal lumen. Penicillin G or metronidazole are recommended only for the treatment of wound botulism [27]. In the case of an antimicrobial therapy for a tetanus infection, the utility of antibiotics is unclear [28]. Antibiotic therapies are not currently recommended for tetanus prophylaxis [29]. However, wounds should be observed for signs of infection, with antibiotics recommended in these instances to prevent bacteria from multiplying. Metronidazole is the most appropriate antibiotic. Parenteral penicillin G is an alternative treatment [29]. Detailed information about the historical aspects and general principles of use of passive immunisation for the treatment of *Clostridium botulinum, Clostridium tetani*, and *Corynebacterium diphtheriae* infections are outlined here. The review mainly focuses on antitoxins that were approved for use by medical agencies many years ago and are still used today. The manuscript also contains information on therapies currently in their research phase that may constitute an alternative to antitoxin treatment in the future. However, none of them have yet been registered for use in humans.

## 2. Botulism

Botulism is an acute paralytic disease caused by a neurotoxin produced by bacteria of the genus *Clostridium*. *Clostridium botulinum* is typically recognised as the primary cause of botulism. Extremely rare neurotoxigenic strains of *Clostridium baratii* type F and *Clostridium butyricum* type E have also been recognised as causative agents of botulism. *C. botulinum* is an obligate anaerobic, spore-forming, gram-positive bacillus that mainly lives in environments that lack oxygen like soil, aquatic sediments, and the gastrointestinal tract of animals [30]. Seven types of *C. botulinum* are known, differing in the type of toxins they produce. Neurotoxin types A, B, E, and F are associated with human diseases [31], while neurotoxin types C, D, and E cause illnesses in other mammals, fish, and birds. The botulinum neurotoxin (BoNT) is a 150 kDa protein and is considered to be the most potent toxin known to man. The lethal dose of the botulinum toxin for humans is approximately 1 ng/kg of body weight [32]. It is estimated that just 1 g of BoNT in aerosol form can cause the death of more than 1.5 million people [33]. BoNT is classified as a biological weapon. According to the Centers for Disease Control and Prevention (CDC), BoNT qualifies as a highly hazardous category A biological agent [34].

BoNT binds to receptors on the presynaptic membrane of the neuromuscular system and, through endocytosis, the 50 kDa light chain of the neurotoxin enters the neuronal cells. Within the axon terminals, BoNT prevents exocytosis by inhibiting the action of the components of the synaptic connection [35]. Due to its protease activity, BoNT inhibits the entry of neurotransmitters (especially acetylcholine) into the synaptic cleft, causing disruption to the functioning of neuromuscular synapses and, subsequently, causing muscle weakness or paralysis. The main forms of botulism in humans are foodborne botulism, infant botulism, wound botulism, and adult intestinal toxemia botulism. Additionally, there are rare case reports of iatrogenic botulism, resulting from therapeutic or cosmetic injections of BoNT, as well as inhalational botulism [36].

In the period of 1976–2006, 524 cases of botulism were reported across twenty-six countries in Europe, Asia, Australia, and the Americas, although it is believed that the actual number of cases may be significantly higher due to underreporting [37]. Globally, the most common form of botulism is foodborne botulism. However, in some countries such as the United States, infant botulism is the predominant form, with over 100 cases recognised annually [38]. According to estimates from the World Health Organization, approximately 475 cases of foodborne botulism are reported annually in Europe, Canada, and the United States. Approximately 15% of all cases result in death, while the majority of the remaining cases lead to disability [39]. Wound botulism and adult intestinal toxemia botulism are the least common, with only a few cases reported each year [40].

BoNT intoxication can occur through various routes. Enteric toxemia botulism in both infants and adults is caused by the ingestion of *C. botulinum* spores, typically found in soil or honey, resulting in the toxin production [41]. Wound botulism occurs after the direct introduction of spores into an open wound, although an increasing number of cases have been reported in individuals who inject intravenous drugs [42]. Foodborne botulism differs from other types of botulism in that it occurs after ingestion of BoNT from contaminated foods. This often occurs in association with the consumption of vacuum-packed products (canned fish, meat) or salted, smoked, or fermented meats, where *C. botulinum* spores experience optimal conditions for growth and toxin production [36]. Typically, the clinical course of botulism (with the exception of wound botulism) begins with the occurrence of nausea and vomiting, although the exact mechanism remains unclear [36]. The initial neurological symptoms include cranial nerve paralysis, which manifests through blurred or double vision, ptosis, photophobia, dysphagia, dysphonia, and dysarthria [35]. Various degrees of muscle paralysis, affecting muscles from the neck to the respiratory system and limbs, have been observed, often leading to permanent deficits. During a botulism outbreak in Thailand, which involved 163 people, 9.3% of the patients experienced limb weakness, while 29.8% of them suffered a paralysis of the respiratory muscles, necessitating mechanical ventilation [43]. The rapidity of the disease onset (ranging from several hours to several days) and the rate of progression primarily depend on the amount of neurotoxin. Death from botulism usually occurs due to respiratory muscle and diaphragm failure [36]. If botulism is suspected, prompt hospital treatment and administration of the botulinum antitoxin are crucial. The mortality rate of untreated botulism is 40% to 50% [35]. Intensive care therapy based on mechanical ventilation improves patient condition, but the most effective treatment remains the administration of the botulinum antitoxin.

### 2.1. Equine Botulinum Antitoxins

The history of antitoxins goes back to the early beginnings of bacteriology. The causative agent of botulism was discovered by the Belgian bacteriologist Émile Pierre-Marie van Ermengem in 1895, and, soon after, the development of an antidote commenced. The first work on the preparation of an anti-botulinum serum was conducted by Kempner in 1897 and then, in 1910, Leuchs made the first attempts at hyperimmunising horses to obtain a serum [44]. The first large-scale research at Lederle Laboratories (currently Pfizer) on equine botulinum antitoxins began in the United States over eight decades ago [44,45]. Equine botulinum antitoxins have been commercially available since the 1960s and are still the only post-exposure products available worldwide to limit the severity of BoNT intoxication [46]. The effectiveness of antitoxins is a result of their ability to neutralise and prevent further internalisation of the toxin. However, there are limitations to antitoxin treatments, as botulinum neurotoxins exert their effects inside the nerve terminal, where they are not susceptible to antibody neutralisation. When symptoms of botulism become apparent, some of the toxin has already been internalised and only some of the circulating toxin can be neutralised. While botulinum antitoxins can neutralise circulating BoNT and prevent the toxin from binding to the neuromuscular junction, they do not reverse the existing paralysis [47]. Almost immediate administration of the antitoxin is crucial to avoid permanent paralysis because the toxin binds irreversibly to the neurons [36]. Unfortunately, botulinum antitoxins are not administered prophylactically but only when botulism is confirmed due to limited access to the product. The production of equine antitoxins is difficult and costly. Limited access is also a consequence of the product’s relatively short shelf life and of the rarity of botulism cases [40].

The botulinum antitoxin contains a mixture of specific immunoglobulins G that bind to specific botulinum toxins and neutralise their toxic properties. Currently, the drug is obtained from the plasma of horses immunised with one of the seven types of BoNT toxoids. Historically, formalin-detoxified toxins were used, while, more recently, recombinant or chemically detoxified toxins have been proposed [48]. In the 1960s, a trivalent ABE equine-derived antitoxin became available and remains widely used. In 2013, a heptavalent (serotypes A, B, C, D, E, F, and G) antitoxin was introduced in the United States. This is the only botulinum antitoxin product licensed for the treatment of non-infant botulism in the US [45]. Other antitoxin combinations are reported, including a quadrivalent ABEF [49], a bivalent AB [50], a monovalent A [51], a monovalent E [52], and a combination of a bivalent AB formulation with a monovalent E formulation [53]. For each antitoxin serotype (A–G), purified F(ab′)_2_/Fab fragments are produced through the pepsin digestion of whole IgG antibodies to remove most of the Fc region, minimising hypersensitivity reactions (despeciation). Despeciation reduces the reactogenicity of the antitoxin, but, unfortunately, also shortens its half-life in the plasma [54]. The shorter plasma half-life of the product may be problematic in cases of wound or intestinal botulism. For instance, in a patient with intestinal colonisation type F botulism, the disease has been shown to initially improve after an antitoxin infusion, only to relapse 10 days later [55]. The probable cause was the short half-life of type F antibodies, while BoNT/F continued to be produced in the intestines. Repeating antitoxin infusions to prevent the relapse symptoms of the intoxication in cases of intestinal colonisation and wound botulism requires greater vigilance [55]. The final antitoxin contains <2% of intact IgG and ≥90% of Fab or F(ab′)_2_ fragments [56]. After obtaining monovalent products, individual antitoxin serotypes are mixed to obtain a polyvalent product. Each monovalent antitoxin pool has failed at cross-neutralising other BoNT toxin types, indicating a high degree of specificity [57]. The nominal established potency values for polyvalent antitoxins are as follows: 7500 international units (IU) anti-A, 5500 IU anti-B, 5000 IU anti-C, 1000 IU anti-D, 8500 IU anti-E, 5000 IU anti-F, and 1000 IU anti-G [54,55]. The amount of IU in the product is sufficient to neutralise the highest BoNT serum levels found in natural disease outbreaks [58]. The botulinum antitoxin is typically administered intravenously in life-threatening cases, though it may sometimes be administered intramuscularly. Due to the possibility of severe allergic reactions, skin sensitivity tests are recommended prior to the administration of the botulinum antitoxin [54].

Many preclinical studies supporting the licensing of botulinum antitoxins have been conducted, including pharmacokinetic, efficacy, and safety assessments in mice, guinea pigs, and rhesus macaques [59,60,61,62,63]. Equine antitoxins have demonstrated high effectiveness in animal studies [64], although their efficacy in humans has never been directly confirmed through double-blind placebo-controlled clinical trials [65]. The heptavalent botulinum antitoxin has been approved by the FDA solely under the animal efficacy rule. This regulation permits the approval of treatments for rare diseases when it is unethical or impractical to conduct randomised human trials, provided their effectiveness is confirmed in animal studies [61,63]. The animal efficacy rule applies only to new medical drugs for which human efficacy studies cannot be conducted because it would be unethical to intentionally expose healthy volunteers to a lethal or highly toxic biological, chemical, nuclear, or radiological substance [66]. Many studies have confirmed that the timing of the antitoxin administration is critical to improve therapeutic outcomes [67]. In one retrospective study involving 134 foodborne botulism patients, those who received the antitoxin within 24 h of symptom onset experienced a lower mortality rate (10%) compared to those treated 24 h after the onset of the symptoms (15%) or to patients who did not receive the antitoxin at all (46%). In addition, early treatment has also been shown to reduce hospitalisation duration and the need for ventilation support [67]. In diagnostic terms, the mouse bioassay (MBA) remains the ‘gold standard’ for the detection of BoNTs. However, in one study, the MBA was found to detect the botulinum toxin in fewer than half of the serum and stool samples examined within 3 days of ingestion (40–44%) and in only 15–23% of the samples collected beyond this period [68]. It is commonly believed that circulating BoNT is cleared from the bloodstream within 1 or 2 days of exposure; however, severe intoxication can result in prolonged clearance time. O’Horo et al.’s manuscript [31] reviewing research on the effectiveness of antitoxin therapies highlighted that the anti-ABE trivalent antitoxin is most commonly reported, with its side effects being less frequently reported than those of other antitoxins. The licensed trivalent ABE antitoxin has been found to be associated with a 9% incidence of hypersensitivity, with nearly 2% incidence of anaphylaxis within 10 min of antitoxin administration and nearly 4% incidence of the serum sickness syndrome [69]. Despite these side effects, the anti-ABE antitoxin has been reported to be well tolerated and highly effective. Reports on the heptavalent antitoxin include two case studies of foodborne botulism outbreaks—one in Thailand [49] and the second in Utah [70]. The low mortality rate in both epidemic outbreaks underscores the antitoxin’s effectiveness [63]. Notably, the proportion of patients requiring mechanical ventilation was significantly reduced among those who received the antitoxin earlier compared to patients who received the antitoxin later. Administration of the antitoxin in the initial stage of symptom manifestation reduced mortality (no deaths) compared to administration in the symptomatic phase of the disease (seven deaths) [63]. The introduction of the botulinum antitoxin has reduced the mortality from botulism in the United States from over 60% in the early 20th century to less than 5% today [35].

### 2.2. Botulism Immune Globulin Intravenous (BIG-IV)

Equine botulinum antitoxins are generally not used for the treatment of botulism in infants due to the possibility of hypersensitivity reactions, including lifelong allergies to horse proteins. Furthermore, equine antitoxins have a short half-life, which may be shorter than the production of toxins in the intestine [35]. To overcome these limitations, a human antitoxin—BIG-IV—was developed in 2003, when the FDA approved the use of human intravenous botulinum immunoglobulin (BIG-IV) for the treatment of infant botulism. It is assumed that both the equine-derived antitoxin and the human-derived immunoglobulin block and inhibit the direct action of BoNT on the presynaptic membrane or nerve terminal [47]. This specific immunoglobulin is classified as an orphan drug. This purified blood immunoglobulin is derived from the pooled plasma of human adult volunteers immunised with a recombinant botulinum vaccine targeting the serotypes A and B (rBV A/B) [71]. Donors are selected based on their high titres of neutralising antibodies against botulinum neurotoxins belonging to types A and B and are tested for blood-borne viruses. To obtain a product suitable for intravenous administration, the collected plasma is fractionated using protein precipitation with cold ethanol, following the Cohn–Oncley method [56]. The final product is a sterile, lyophilised IgG powder treated with a solvent and stabilised with 5% sucrose and 1% albumin, both free from preservatives. To date, this product has been administered to more than 2180 American infants with botulism, resulting in an average hospital stay reduction of 3.6 weeks and a cost saving exceeding $174 million [56]. BIG-IV contains ≥15 IU of antibodies against BoNT/A and ≥4 IU of antibodies against BoNT/B. Its efficacy was demonstrated in a 5-year double-blind, randomised, placebo-controlled study in California and a 6-year nationwide open-label study [60]. In both studies, treatment with BIG-IV significantly reduced hospitalisation times, including the need for intensive care. No serious side effects were observed. It was found that BIG-IV has a significantly lower risk of anaphylaxis compared to trivalent equine antitoxins [72,73]. Since BIG-IV is derived from human plasma, it has a prolonged circulation time (with an average serum half-life of approximately 28 days), providing extended protection during the intestinal colonisation by *Clostridium* sp. [45]. A human-derived product such as BIG-IV would be desirable for the initial treatment and prevention of botulism [73].

### 2.3. Recombinant Monoclonal Antibodies (mAbs)

BIG-IV is derived from hyperimmunised human donors, with the acquisition of such large amounts of antitoxins posing challenges due to the limited number of donors. For this reason, access to the product, as in the case of equine botulinum antitoxins, remains restricted. Recombinant mAbs could be an alternative to equine and human antitoxins that would constitute a source of antitoxins in unlimited quantities, without the risk of transmitting infectious diseases. Additionally, sequence variations among BoNT subtypes must be made when generating new neutralising antibodies [30]. To achieve better specificity, Chen and colleagues constructed a BoNT epitope binding model in 1997 to aid in the design of neutralising antibodies. It was also demonstrated that clostridial haemagglutinins protect BoNT against proteolysis [74]. Fifteen antibodies against BoNT were identified that bind to the catalytic domain at conformational epitopes. Based on this mapping of antibodies across different domains of BoNT, a model of the BoNT complex was proposed [74]. Furthermore, Adekar and colleagues examined the native human antibody response to BoNT [75]. Using primary human B cells and a murine cell line, they generated hybridomas secreting specific IgM antibodies binding to BoNT/A. One of these antibodies fully neutralised a lethal dose of BoNT/A in vivo. Subsequently, by using peripheral blood B cells, they generated libraries of stable hybridomas secreting antigen-specific human IgG antibodies [75]. Mouse mAbs against BoNT/A, B, E, and F have been described [76,77]. These mAbs exhibit neutralising properties in vivo, when administered individually and in combination. However, the combination of multiple antibodies has been found to neutralise a significantly larger dose of the BoNT [78]. Studies have also reported six highly protective mAbs from sheep vaccinated with BoNT/A1 toxoids. Their trivalent combination has been shown to be 100% protective against clinical symptoms and death caused by botulism [79]. Results of numerous studies demonstrate that mAbs can be used to develop humanised therapeutic antibodies. Monoclonal Ab for BoNTs belonging to the serotypes B and E are in advanced preclinical studies [80], while studies of antibodies for BoNTs belonging to the serotypes C and D have recently undergone preliminary preclinical testing. Antibodies against BoNT serotypes F and G are still under evaluation [80]. mAbs are more potent than equine-derived antitoxins and exhibit a half-life time of approximately 1 month [80]. The half-life of equine antitoxins belonging to types A, B, and E has been estimated at 6.5 days, 7.6 days, and 5.3 days, respectively, in a single patient [81]. Due to the absence of heterologous antigens, mAbs are expected to cause fewer side effects. Recombinant mAbs appear to be highly promising for the new generation of antitoxin production to combat BoNT intoxication. They offer a stable source of antitoxins, overcoming the challenges associated with current products.

### 2.4. Generation of BoNT-Neutralising Antibodies by Phage Display Technology

In 1997, Amersdorfer and colleagues first described antibodies capable of neutralising BoNT/A that had been produced using phage antibody libraries. From the spleens of mice vaccinated with the carboxylic domain of the BoNT heavy chain (BoNT/A HC), they isolated antibodies for the generation of scFv phage antibody libraries constructed from the Ig heavy-chain and kappa light-chain variable region genes. The results of this research suggest the presence of two binding sites on BoNT/A HC involved in toxin internalisation and toxicity [82]. The neutralising scFv antibodies binding to both these epitopes were also screened by use of a phage display random peptide library. Mapping of scFv antibodies binding to BoNT/A HC epitopes revealed a limited number of scFvs recognising the toxin epitopes. Interestingly, it was observed that the antibody-producing cell clones from the library of non-immunised people were not the same as those from the immunised volunteers. This suggests that the pentavalent botulinum toxoid vaccine directs the humoral immune response to a limited number of epitopes in the HC binding domain [82]. The immunization of mice with the selected phage clones from naive libraries has been found to induce a specific humoral response against BoNT/A. These findings demonstrate that phage-displayed random peptide libraries are valuable tools for identifying neutralising epitopes and potential vaccine candidates [83]. The use of naive human scFv phage display libraries to select human-specific neutralising antibodies by mapping epitopes against BoNTs represents a promising therapeutic approach to developing effective human antibodies [83]. A library of yeast-displayed BoNT/A HC mutants has been used for the construction of epitopes for three neutralising BoNT/A mAbs and for the examination of antibody affinity maturation [84]. Yeast-display scFv antibodies constructed from immunised mice or humans have also been employed in research for cross-toxinotype reactivity [85]. Immune phage-display libraries of macaque origin (*Macaca fascicularis*) have been utilised to select recombinant antibodies neutralising BoNT/A, B, and E with ex vivo and in vivo cross-neutralising protection [48,57]. The three-antibody combination (NTM-1633), generated from libraries of yeast-displayed scFv antibodies created from variable gene regions of humans immunised with pentavalent toxoids and the BoNT/E-binding scFv, is in preclinical development for the neutralisation of BoNT/E1, BoNT/E3, and BoNT/E4 [86]. XOMA3AB, developed in the United States from a human phage library, is a mixture of three IgGs that bind different regions of BoNT/A [87]. Phase I clinical trials of XOMA3AB have been successful [88]. Several reports on the use of camelid VHHs (nanobodies) to inhibit the activity of BoNTs have been described [89,90,91,92]. Lam et al. developed VHH antibodies targeting the protease domains of BoNT/A and BoNT/B using a phage display library. Then, by using a biochemical assay and X-ray crystallography, they investigated the structures and inhibition mechanisms of several VHHs that recognise highly conserved epitopes across BoNT/A and BoNT/B subtypes [89]. Godakova et al. selected 15 specific BoNT/A-neutralising alpaca VHHs from a VHH antibody immune library through the implementation of the phage display method. Two clones were fused with human IgG Fc fragments (VHH-Fc). The fusion bodies greatly increased their in vivo neutralising potency on mice [92].

The phage display method represents a new approach to the development of novel treatments for botulism. This technique offers numerous advantages, including speed, specificity, and potentially safer alternatives to traditional therapies. However, challenges related to the diversity of toxin serotypes must be addressed before these therapies can be fully integrated into clinical practice. Recent reports on new BoNT types have been published, including on the hybrid toxin type A/F [93,94] and a newly identified toxin based on the gene sequence of *C. botulinum* isolates [95]. These reports illustrate important challenges in the field of botulism research, as the discovery of clinically significant new botulinum toxins remains a possibility. Consequently, continued research into potential antidotes is crucial to address these evolving threats.

## 3. Tetanus

Tetanus is a potentially fatal infectious disease caused by toxins produced by strains of *Clostridium tetani*. The bacterium *C. tetani* was first isolated from a human by the Japanese bacteriologist Shibasaburo Kitasato in 1889 [96]. *C. tetani* is a gram-positive, spore-forming, motile, anaerobic bacillus. Its spores are widely distributed in the soil and in human and animal faeces, where they can remain viable in the environment for many years. *C. tetani* produces tetanospasmin, also referred to as the tetanus toxin (TeNT), a neurotoxin responsible for the clinical manifestations of tetanus [29]. The tetanus toxin is one of the most potent toxins known by weight. It is synthesised as a 150 kDa single-chain protein and undergoes post-translational modifications to form the active toxin [97]. The active form of the tetanus toxin (TeNT) consists of an N-terminal light chain (TeNT-L, ~50 kDa) and a C-terminal heavy chain (TeNT-H, ~100 kDa), linked through a disulfide bond. The heavy chain contains two functional domains: the N-terminal translocation domain (TeNT-HN, ~50 kDa) and the C-terminal receptor-binding domain (TeNT-Hc, ~50 kDa) [98]. The TeNT-Hc domain has been shown to act as a protective antigen, and immunisation with TeNT-Hc alone has been found to provide immunity comparable to that obtained with full-length tetanus toxoids [99]. The estimated lethal dose of the tetanus toxin for humans is 0.2 ng/kg of body weight [97]. Spores of *C. tetani* enter the body through contaminated wounds and, under anaerobic conditions, germinate into vegetative forms of bacilli, producing and releasing the tetanus toxin. The toxin spreads through the blood and lymphatic systems, acting on several sites within the central nervous system. The typical clinical symptoms of tetanus appear when the toxin inhibits the release of the inhibitory neurotransmitters, resulting in painful muscle spasms [29].

Tetanus is the only vaccine-preventable disease that is not contagious [100]. Despite the long-term availability of an effective vaccine, the high mortality rates associated with tetanus contribute to the fact that tetanus remains a significant public health problem in many parts of the world. The tetanus mortality rate in 2019 was about 54% in low sociodemographic index (SDI) regions and 35% in high SDI regions [101]. The introduction of the tetanus toxoid vaccine has resulted in a significant decline in tetanus incidence worldwide since the mid-20th century. Today, tetanus primarily affects low-income countries or regions with low vaccination rates and unclean birthing practices [102]. Most reported cases of tetanus worldwide occur among newborns and mothers who have not been appropriately vaccinated with vaccines containing tetanus toxoids [103]. According to WHO data, approximately 25,000 newborns died from neonatal tetanus in 2018 [102]. Tetanus-related deaths worldwide have decreased dramatically, from an estimated 275,000 in 1990 to 35,000 in 2019 [101].

The purposes of tetanus therapies include inactivating the circulating toxins, preventing further toxin production by eradicating the bacterium *C. tetani* from the wound site, and providing supportive care for the duration of the illness [104]. Equine tetanus antitoxins or human tetanus immunoglobulins can be used to neutralise the circulating tetanus toxin and should be administered as soon as possible before the toxin reaches the nervous system [104].

### 3.1. Discovery of Tetanus Antitoxin

In 1890, Emil von Behring and Shibasaburo Kitasato published their discoveries on the transmission of immunity against diphtheria and tetanus through serum administration in animals. They demonstrated the effectiveness of serotherapy at treating both diphtheria and tetanus. They used the term “antitoxins” to describe the sera from immune animals which contained substances capable of neutralising toxins [29,105]. Behring and Kitasato demonstrated that immunity to high doses of the tetanus toxin can be achieved by repeatedly injecting animals with increasing sublethal doses of the purified toxin. They also showed that transferring the blood of tetanus-immune rabbits into naive mice conferred complete protection against a normally lethal dose of virulent *C. tetani* or of the tetanus toxin. It was the first example of the application of passive immunisation for the effective treatment of tetanus [105].

The horse became the preferred animal for the production of tetanus antitoxins. In 1895, the French veterinarian Edmond Nocard reported the successful use of serum therapy in the treatment of horses suffering from tetanus [106]. He demonstrated that the prophylactic use of the anti-tetanus serum was highly effective. Nocard studied the preventive use of tetanus antitoxins in the suburbs of Paris, where tetanus outbreaks were common, and showed that the antitoxins prevented tetanus if administered before a planned surgery or after an accidental injury. In areas with high tetanus prevalence, veterinarians started recommending the immediate injection of antitoxins into animals in cases of wounds susceptible to tetanus [107].

For the tetanus treatment to be effective, it is essential that the antitoxin be administered as early as possible. Delayed administration, after the onset of the symptoms, is significantly less effective in both animal models and humans [108,109]. This is due to the fact that the disease directly depends on the presence of tetanus toxins in the central nervous system [106]. The efficacy of the equine anti-tetanus serum was confirmed during World War I, when soldiers faced heightened exposure to tetanus. Tetanus soon became a medical priority, prompting the immediate introduction of broad-scale anti-tetanus serotherapies for wounded soldiers. The systematic use of the prophylactic anti-tetanus serum radically reduced the incidence of tetanus among wounded soldiers. The widespread administration of equine anti-tetanus serum proved crucial and saved countless lives [110]. The equine tetanus antitoxin has prevented life-threatening tetanus infections in hundreds of thousands of injured individuals, making it one of the most effective preventive interventions in wartime medicine [111].

### 3.2. Production of Equine Tetanus Antitoxins

Over time, various production methods for equine tetanus serum have been developed. Behring and Kitasato, who were pioneers in this field, initially immunised horses by injecting them with a tetanus toxin that had been previously weakened chemically, primarily various iodine combinations. When the horses had achieved a certain degree of immunity, they were injected with gradually increasing doses of the unattenuated toxin. After a period, the horse’s serum was tested to determine whether it contained sufficient amounts of the antitoxin. An alternative immunisation schedule involved an initial injection of a toxin–antitoxin mixture, followed by separate injections of either the pure toxin or the combination of toxin and antitoxin [112].

The discovery of anatoxin in 1923 by the French veterinarian Gaston Ramon revolutionised the production of equine tetanus antitoxins. A formalin treatment converted thetoxin into anatoxin, a form that was virtually non-toxic but retained its antigenic properties [99,113]. In 1925, Ramon observed that induced inflammatory infiltration in the horse influenced the production of high-titre serum. Research conducted by Ramon has demonstrated that inflammation induced by the co-administration of various chemicals, such as oils and extracts of tapioca, stimulates the production of antitoxins in horses [114].

Further modifications to Ramon’s immunisation method were explored to enhance the production of anti-tetanus serum. One approach involved injecting an emulsion of olive oil, lanolin, and the tetanus toxin or anatoxin to induce an inflammatory infiltrate. Three or four days later, the tetanus toxin was injected into the already existing inflammatory infiltrate. This procedure made it possible to obtain antitoxins with titres of up to 4000–4800 IU per cm^3^ [112].

The production of equine tetanus antitoxins has evolved considerably since the early 20th century. The administration of equine tetanus antitoxins has been frequently associated with adverse reactions, including anaphylaxis and serum sickness, partly due to residual plasma proteins other than immunoglobulins that remained after serum fractionation [104]. In order to reduce the antigenicity of equine tetanus antitoxins intended for use in humans, the proteolytic treatment was introduced during the manufacturing process, resulting in cleaved Fab fragments that retained their neutralising activity [115]. Further advancements include the development of manufacturing techniques to purify equine IgG antitoxins and the incorporation of pasteurisation steps to reduce the risk of virus transmission [116]. Despite these improvements, equine antitoxins provide only short-term passive immunity. The half-life of refined equine antitoxins in humans is less than 2 weeks and the protective levels are detected only for about 4 weeks [116,117].

### 3.3. Human Tetanus Immunoglobulin (TIG)

Compared to equine antitoxins, the human tetanus immunoglobulin (TIG) persists in the serum for a much longer time. The half-life of tetanus antibodies from TIG in adults is 4–5 weeks, but the protective levels (0.01 units per mL) are maintained for up to 14 weeks [117]. Introduced in the early 1960s, TIG is produced through cold-ethanol fractionation of the plasma from healthy volunteers specifically immunised against tetanus. The frequency of local and systemic reactions associated with TIG administration is much lower than in the case of equine antitoxins [118]. Furthermore, its human origin allows TIG to be used directly without requiring a prior skin test, unlike equine antitoxins which carry a higher risk of adverse reactions [119]. Due to its higher safety profile and the longer half-life, TIG has quickly become the standard treatment for tetanus in high-income countries. However, its high cost and limited availability in lower-income regions often result in equine antitoxins being more commonly used [120].

According to WHO guidelines, if tetanus is suspected, a single intramuscular dose of tetanus human immunoglobulin TIG is recommended as soon as possible to prevent further progression of the disease. If TIG is not available, equine tetanus antitoxins can be administered intravenously after testing for hypersensitivity. Intravenous immunoglobulin (IVIG) may also serve as an alternative [103].

TIG should be administered promptly upon diagnosis to neutralise the circulating toxin before it reaches the nervous system [28,121]. The optimal dose of TIG for the treatment of tetanus has not been definitively established. When TIG was introduced to treat tetanus in the 1960s, a dosage ranging from 3000 units to 6000 units was recommended based on calculations of antibody levels required to provide minimal protection against the effects of the tetanus toxin [122]. Despite limited evidence supporting this dosage range, it became the standard in relation to TIG therapy administered intramuscularly in a single injection [111,123]. Subsequent retrospective analyses of tetanus cases reported in the USA between 1965 and 1971 have found that a TIG dosage of 500 units is as effective as higher doses at reducing mortality [123]. Lowering the dosage of TIG to 500 units not only reduced the injection volume of the antitoxin, but also minimised painful stimuli, which could trigger spasms in patients suffering from tetanus. Currently, a 500-unit dose of TIG is commonly recommended [28,121,124], although some authorities continue to prefer the higher range of 3000–6000 units [125,126,127].

The first controlled double-blind trial [117] compared equine antitoxins (10,000 units) and human immunoglobulin (500 units) in cases of tetanus neonatorum, with two treatment groups of 65 infants suffering from tetanus neonatorum. The study found no significant difference in treatment efficacy between the two groups. Importantly, no adverse reactions were attributed to either equine antitoxins or TIG in a study conducted on 130 infants [117].

A number of current studies have explored the potential benefits of administering TIG or equine antitoxins intrathecally, rather than intravenously or intramuscularly. However, this method remains controversial, as research findings on its effectiveness are conflicting [65,128,129,130]. Antibody preparations administered systemically do not cross the blood–brain barrier; intrathecal administration theoretically allows for the neutralisation of the extracellular toxin in the central nervous system, as it moves through the synaptic spaces [104]. Several studies have shown the potential advantages of intrathecal TIG administration, such as a significant reduction in mortality and a significant improvement of clinical course and shortened duration [131,132,133]. However, two published meta-analyses have provided conflicting conclusions on whether intrathecal administration is beneficial [128,130]. In a recent randomised, controlled trial, intrathecal administration of tetanus antitoxins has been shown to be safe, but it has also been demonstrated that it is not associated with an overall benefit in the treatment of tetanus compared with intramuscular antitoxin administration [65].

### 3.4. TeNT Neutralising Antibodies Generated by Phage Display Technology

Phage display technology is a new strategy for obtaining effective tetanus-neutralising antibodies. Among the tetanus-neutralising antibodies, only few nanobodies have been developed. Rossotti et al. [134] showed that the tetanus-neutralising capacity of nanobodies from llamas was drastically improved following their fusion to a second nanobody specific to the complement receptor CD11b/CD18 (Mac-1), enabling the mice to survive a 10-fold more lethal dose of the tetanus toxin [134]. In the study performed by de Smit et al., llama-derived nanobody multimers were developed. As building blocks to generate 11 nanobody multimers, four nanobody monomers binding to TeNT with high affinity, covering various antigenic domains, and which included three monomers that inhibited TeNT binding to neuron gangliosides were chosen. These multimers consisted of either one or two different TeNT-binding nanobodies fused to one nanobody binding to either albumin (A12) or immunoglobulin (G13) to extend the serum half-life in animals. Multimers containing two TeNT-binding nanobodies demonstrated more than a 10-fold increase in affinity when compared to multimers containing only one TeNT-binding nanobody [135]. A study performed by Cheng et al. demonstrated a promising use of phage display technologies for the development of tetanus-neutralising antibodies [99]. The research constructed a phage display nanobody library by immunising a camel with the TeNT-Hc domain as the antigen. Fourteen anti-tetanus nanobodies were obtained, which demonstrated that the TeNT-Hc domain is an effective immunogen capable of generating toxin-neutralising antibodies. Upon fusing the anti-tetanus nanobodies with the Fc fragment from the human antibody, 11 chimeric heavy-chain antibodies exhibited nanomolar binding affinity with the TeNT-Hc domain. Among these, three antibodies completely neutralised TeNTs and demonstrated potential for both prophylactic and therapeutic uses. The evaluation of the protective, neutralising efficacy demonstrated that low concentrations of these antibodies are able to completely protect mice against 20 × LD_50_ of tetanus toxins, with a neutralising potency of 0.2–0.4 IU/mg. Moreover, the antibodies showed both prophylactic and therapeutic efficacy against TeNT exposure in a mouse model [99].

## 4. Diphtheria

Diphtheria is caused by an exotoxin produced by the gram-positive bacteria *Corynebacterium diphtheriae*, *C. ulcerans*, and *C. pseudotuberculosis*, encoded by a bacteriophage integrated into the bacterial chromosome [136,137]. Transmission occurs through droplets or direct contact with secretions from or the belongings of an infected individual. The bacteria usually enter the body through the nasopharynx, colonising the mucous membranes of the upper respiratory tract, or, less commonly, through damaged skin or the mucous membranes of other areas (e.g., genitals, conjunctiva). Initial diphtheria symptoms are nonspecific and gradually intensify and include weakness, fever, sore throat, headache, muscle pain, hoarseness, and cough. In advanced stages, erythematous changes develop in the mucous membrane of the throat up to the trachea. As a consequence, white or greyish deposits and necrotic pseudomembranes are formed and adhere tightly to the mucous membranes, with their separation often resulting in bleeding [138]. The toxin produced by corynebacteria spreads through the bloodstream, damaging the heart, kidneys, liver, and central nervous system [139]. The toxin, which inhibits protein synthesis, can cause acute demyelinating polyneuropathy, with symptoms such as difficulty swallowing (due to nerve demyelination in the throat) or motor weakness and autonomic dysfunction, resulting from the involvement of the central nervous system. The lethal dose of this toxin in humans is estimated at approximately 0.1 μg/kg [140].

The most effective preventive method is vaccination with a diphtheria toxoid, an inactivated form of the toxin [141]. Although vaccinated individuals can also get sick, the risk of infection is significantly lower, and the symptoms are less severe. The introduction of vaccinations has led to a significant decrease in the number of cases, particularly in countries with robust immunisation programmes. However, cases continue to emerge in endemic regions, where the vaccination programme is still poorly implemented (e.g., Algeria, Egypt, Brazil, Colombia, Ecuador, the Dominican Republic, Afghanistan, China, India, Indonesia, Thailand, the Philippines, Iran, Iraq, and Syria) [142,143]. In Europe, the annual number of diphtheria cases ranged between 20 and 66 in 2011–2021 but increased dramatically to 359 cases in 2022 and 171 cases in 2023. According to WHO data, the number of diphtheria cases globally varied between 7102 and 24,778 cases annually from 2016 to 2023 [144].

The basis for diagnosing diphtheria is the isolation of bacteria from a throat swab. However, when clinical suspicion is high, treatment should commence immediately without awaiting bacteriological confirmation. Administering antitoxins as the primary treatment is crucial for improving patient outcomes [145]. In addition to the antitoxins, antibiotics (erythromycin, azithromycin, penicillin) and supportive care (nonsteroidal anti-inflammatory drugs, removal of pseudomembranes) are used as part of anti-diphtheria therapies [146,147]. The mortality rate associated with diphtheria is estimated at 5–10%; however, in children under five years of age and adults over 40 years of age, the mortality rate may increase to 20% [148].

### 4.1. Equine Diphtheria Antitoxin

Horse serum therapy, commonly known as diphtheria antitoxin (DAT), was introduced in 1890, when Behring and Kitasato proved that passive immunisation with anti-tetanus and anti-diphtheria sera could protect against these bacterial diseases [105]. In 1894, a large-scale clinical trial of DAT therapy was conducted in Paris, providing clear evidence in support of the effectiveness of the antitoxin by comparing mortality rates across groups of children treated with and without it [149,150]. Horse serum therapy was a breakthrough in the treatment of diphtheria, especially in relation to children, and was awarded the first Nobel Prize in Medicine in 1901.

DAT is a solution of concentrated globular proteins containing antibodies obtained from the serum of horses hyperimmunised with the diphtheria toxoid and toxin. However, this serum contains a large and varied number of different types of antibodies of unknown specificity. DAT products, some of which are produced as F (ab’)_2_ following pepsin digestion, are in clinical use today for diphtheria therapy and outbreak management [151,152]. Due to the nature of the product, there is a possibility of differences in quality between batches [153]. Moreover, the administration of DAT is associated with a significant risk of anaphylactic response or acute and delayed hypersensitivity. The literature describes cases where antibodies against foreign antigens introduced during the administration of animal sera formed immune complexes that can precipitate in joints or small vessels, initiating a serious inflammatory response known as serum sickness [154]. DAT is currently difficult to access for patients due to the cessation of production in many countries (e.g., Poland, France, Spain, Portugal, Italy, Sweden, and Norway). Efficient supplies of DAT are increasingly at risk due to the limited number of manufacturers [151].

### 4.2. Human Anti-Diphtheria Immunoglobulins and Monoclonal Antibodies

Given the multifactorial challenges faced by horse serum therapy and the continued prevalence of the disease, there is an urgent need to find an alternative to DAT. The use of antitoxins derived from human rather than equine blood would be a more satisfactory solution, since allergic reactions to human antibodies are much less common. Unfortunately, this therapy presents major limitations. It is estimated that the quantity of antibodies required to combat diphtheria ranges from 5000 IU to 50,000 IU. Experiments with donors with high antibody titres (levels of approximately 3 IU/mL) indicate that, even after an enriching preparation with antitoxins derived from human plasma, the resulting therapeutic product would contain a maximum of 50 IU/mL. Consequently, an effective treatment would require unreasonably large doses, in the order of thousands of ml [155,156].

The technology behind the production of monoclonal antibodies has greater potential, especially since the diphtheria exotoxin is highly conserved. This increases the likelihood of monoclonal antibodies being highly effective at neutralising the toxin. In 2006, Kakita et al. [157] isolated a human monoclonal antibody against the diphtheria toxin (DT) which could bind to the B fragment of the toxin. The neutralising activity was assessed in a rabbit skin test, although no further studies on this particular antibody have since been published. A more recent study, published in 2013, demonstrates the high effectiveness of another monoclonal antibody, the human IgG_1_ mAb (designated S315), at preventing the toxin from binding to the diphtheria receptor, a heparin-binding epidermal-growth-factor-like growth factor [158]. The relative potency of the S315 mAb was established by comparing its neutralising capacity with that of the equine polyclonal DAT standard in a guinea pig model of the disease [159]. In 2019, the S315 mAb was advanced to phase 1 clinical trials [160].

### 4.3. Recombinant Human Antibody Fragments for the Diphtheria Toxin

More recently, the implementation of phage display technology has been explored as a means of developing alternative antibody treatments for diphtheria. By using this technology, Khalili et al. [161] constructed a human single-chain fragment variable (HuscFv) library from B cells of volunteers immunised with a diphtheria vaccine. From this library, antibody clones which showed positive reactivity to the diphtheria toxin in ELISA and a toxin neutralisation capacity in a Vero cell assay were identified. The selected HuscFv clones revealed a neutralising activity ranging from 0.6 µg to 1.2 µg against a double cytotoxic dose of the diphtheria toxin, making them a promising alternative to horse serum diphtheria antitoxins. However, further extensive studies on HuscFv are necessary.

Studies on antibodies belonging to IgG subclasses isolated from a camel vaccinated against diphtheria and tetanus reveal a significantly higher efficiency on the part of the nanobodies fraction in binding to the diphtheria toxin antigen compared to conventional antibodies [162]. Recombinant nanobodies capable of inhibiting a proprotein convertase, i.e., furin, whose function leads to the activation of many proteins (including the diphtheria toxin), also have a high therapeutic potential. It has been shown that the purified fraction of these nanobodies, acting as specific non-competitive furin inhibitors, effectively inhibits the cleavage of the diphtheria toxin into its enzymatically active fragment A and protects cell cultures from diphtheria-toxin-induced cell toxicity [163]. However, although these results are very encouraging for anti-diphtheria therapy, further clinical studies are necessary for final verification.

## 5. Conclusions

Passive immunisation has been utilised in clinical practice since the late 19th century. The active components of the gamma globulins, which protect against the lethal effects of botulinum, diphtheria, and tetanus toxins, are immunoglobulins, commonly known as antibodies. However, the limited availability and potential side effects of currently approved immunoglobulin therapies have created an urgent need for innovative medicinal products. While equine-derived antitoxins have proven to be effective, they suffer from a short half-life, and disease recurrence remains a concern. Recombinant antibodies and their fragments offer a promising alternative, as their half-life can be extended. Novel therapies represent a promising therapeutic option. They offer a greater specificity and reduced side effects compared to animal-derived antitoxins, such as hypersensitivity allergic reactions. However, challenges such as regulatory obstacles still exist. If research progresses successfully, modern antibodies could become an important tool in the treatment of many diseases. Novel approaches, such as phage display techniques, are aimed at developing more specific and better tolerated antibodies. The production of antibodies through the phage library technique is much faster and cheaper. However, most of these products remain in the experimental or preclinical stages requiring significant further research to transition into clinical use. Currently available ‘antitoxins’ offer treatment options; however, they have notable limitations. Primarily, they act only on circulating toxins and block the binding of the toxin to its receptors. These antibodies cannot directly inhibit the proteolytic activity of the toxin that has already entered the cell. For the most effective outcome, the antitoxin must be administered in the earliest phase of the disease. Due to the above, while passive immunisation may be sufficient for the prophylactic or therapeutic treatment of acute infections or disease remissions, long-term immunity requires active immunisation. Highly effective vaccines protecting against diphtheria and tetanus are widely used. Unfortunately, the vaccination rates have been decreasing in recent years [164]. Several vaccine candidates against botulism are in preclinical or clinical development [71]. To provide access to BIG-IV, the safety and efficiency of the recombinant rBV A/B vaccine has had to be demonstrated in a phase 2b clinical study [50], with the vaccine currently under FDA licensure [165]. Botulinum toxoid vaccines have been produced and used in Japan [166]. The decline in public confidence in vaccinations has become a significant challenge in recent years [167]. Several factors have an impact on this trend, including misinformation, false claims spread through social media, and political and cultural controversies surrounding vaccination. An example of this is the growing number of people who doubt the safety of vaccines or fear potential side effects, despite evidence of their effectiveness and safety [167]. Research by UNICEF has shown that, since the onset of the COVID-19 pandemic, confidence in childhood vaccines in Europe and Central Asia has decreased by more than 10 percentage points [168]. Additionally, reports from various countries indicate the increasing influence of misinformation on people’s health decisions, making it harder to achieve high vaccination rates [167].

On the other hand, continued research into the development of antibody-based treatment therapies is crucial, particularly in light of the emergence of antibiotic-resistant bacteria and pathogens that secrete antigenically altered virulence factors.

## References

[1] Graham B.S., Ambrosino D.M. (2015). History of Passive Antibody Administration for Prevention and Treatment of Infectious Diseases. Curr. Opin. HIV AIDS.

[2] Winau F., Winau R. (2002). Emil von Behring and Serum Therapy. Microbes Infect..

[3] Marano G., Vaglio S., Pupella S., Facco G., Catalano L., Liumbruno G.M., Grazzini G. (2015). Convalescent Plasma: New Evidence for an Old Therapeutic Tool?. Blood Transfus..

[4] Cohn E.J., Strong L.E., Hughes W.L., Mulford D.J., Ashworth J.N., Melin M., Taylor H.L. (1946). Preparation and Properties of Serum and Plasma Proteins. IV. A System for the Separation into Fractions of the Protein and Lipoprotein Components of Biological Tissues and Fluids1a, b, c, d. J. Am. Chem. Soc..

[5] Stokes J., Maris E.P., Gellis S.S. (1944). Chemical, Clinical, and Immunological Studies on the Products of Human Plasma Fractionation. XI. The Use of Concentrated Normal Human Serum Gamma Globulin (Human Immune Serum Globulin) in the Prophylaxis and Treatment of Measles. J. Clin. Investig..

[6] Llewelyn M.B., Hawkins R.E., Russell S.J. (1992). Discovery of Antibodies. BMJ.

[7] Karpas A., Hill F., Youle M., Cullen V., Gray J., Byron N., Hayhoe F., Tenant-Flowers M., Howard L., Gilgen D. (1988). Effects of Passive Immunization in Patients with the Acquired Immunodeficiency Syndrome-Related Complex and Acquired Immunodeficiency Syndrome. Proc. Natl. Acad. Sci. USA.

[8] Lasocka J., Bielawski A., Lachert E. (2021). Passive Immunization in the Combat against Infectious Diseases (COVID-19 Included). J. Transfus. Med..

[9] Lachert E., Kubis J., Antoniewicz-Papis J., Rosiek A., Woźniak J., Piotrowski D., Przybylska Z., Mikołowska A., Marschner S., Łętowska M. (2018). Quality Control of Riboflavin-Treated Platelet Concentrates Using Mirasol^®^ PRT System: Polish Experience. Adv. Clin. Exp. Med..

[10] Radosevich M., Burnouf T. (2010). Intravenous Immunoglobulin G: Trends in Production Methods, Quality Control and Quality Assurance: Intravenous Immunoglobulin G. Vox Sang..

[11] Abolhassani H., Asgardoon M.H., Rezaei N., Hammarstrom L., Aghamohammadi A. (2015). Different Brands of Intravenous Immunoglobulin for Primary Immunodeficiencies: How to Choose the Best Option for the Patient?. Expert Rev. Clin. Immunol..

[12] Stępień A., Korsak J., Kozubski W., Ryglewicz D., Losy J., Drozdowski W., Kotowicz J., Nyka W., Kwieciński H. (2011). Stanowisko grupy ekspertów dotyczące stosowania dożylnych immunoglobulin w leczeniu chorób układu nerwowego. Neurol. Neurochir. Pol..

[13] Arumugham V.B., Rayi A. (2024). Intravenous Immunoglobulin (IVIG). StatPearls.

[14] Epland K., Suez D., Paris K. (2022). A Clinician’s Guide for Administration of High-Concentration and Facilitated Subcutaneous Immunoglobulin Replacement Therapy in Patients with Primary Immunodeficiency Diseases. Allergy Asthma Clin. Immunol..

[15] Sullivan D.J., Gebo K.A., Shoham S., Bloch E.M., Lau B., Shenoy A.G., Mosnaim G.S., Gniadek T.J., Fukuta Y., Patel B. (2022). Early Outpatient Treatment for COVID-19 with Convalescent Plasma. N. Engl. J. Med..

[16] Freysd’ottir J., George A.J.T., Urch C.E. (2000). Production of Monoclonal Antibodies. Diagnostic and Therapeutic Antibodies.

[17] Beaber J.W., Tam E.M., Lao L.S., Rondon I.J. (2012). A New Helper Phage for Improved Monovalent Display of Fab Molecules. J. Immunol. Methods.

[18] Posner J., Barrington P., Brier T., Datta-Mannan A., Barrett J.E., Page C.P., Michel M.C. (2019). Monoclonal Antibodies: Past, Present and Future. Concepts and Principles of Pharmacology.

[19] Motilal S., Mishra S., Arjun M., Venkatesh M.P. (2024). Regulatory Challenges and Landscapes of Monoclonal Antibody Registration: Global Outlook. Int. J. Pharm. Pharm. Sci..

[20] Jin B., Odongo S., Radwanska M., Magez S. (2023). NANOBODIES^®^: A Review of Diagnostic and Therapeutic Applications. Int. J. Mol. Sci..

[21] Hamers-Casterman C., Atarhouch T., Muyldermans S., Robinson G., Hammers C., Songa E.B., Bendahman N., Hammers R. (1993). Naturally Occurring Antibodies Devoid of Light Chains. Nature.

[22] Harmsen M.M., De Haard H.J. (2007). Properties, Production, and Applications of Camelid Single-Domain Antibody Fragments. Appl. Microbiol. Biotechnol..

[23] Wu Y., Jiang S., Ying T. (2017). Single-Domain Antibodies As Therapeutics against Human Viral Diseases. Front. Immunol..

[24] Arbabi-Ghahroudi M. (2017). Camelid Single-Domain Antibodies: Historical Perspective and Future Outlook. Front. Immunol..

[25] Amersdorfer P., Marks J.D. (2000). Phage Libraries for Generation of Anti-Botulinum scFv Antibodies. Bacterial Toxins.

[26] Farizo K.M., Strebel P.M., Chen R.T., Kimbler A., Cleary T.J., Cochi S.L. (1993). Fatal Respiratory Disease Due to Corynebacterium Diphtheriae: Case Report and Review of Guidelines for Management, Investigation, and Control. Clin. Infect. Dis..

[27] Yutani M., Matsumura T., Fujinaga Y. (2021). Effects of Antibiotics on the Viability of and Toxin Production by *Clostridium botulinum*. Microbiol. Immunol..

[28] Nath A. (2015). Infections of the Nervous System. Clin. Infect. Dis..

[29] CDC Chapter 21: Tetanus. https://www.cdc.gov/pinkbook/hcp/table-of-contents/chapter-21-tetanus.html.

[30] Peck M., Smith T., Anniballi F., Austin J., Bano L., Bradshaw M., Cuervo P., Cheng L., Derman Y., Dorner B. (2017). Historical Perspectives and Guidelines for Botulinum Neurotoxin Subtype Nomenclature. Toxins.

[31] O’Horo J.C., Harper E.P., El Rafei A., Ali R., DeSimone D.C., Sakusic A., Abu Saleh O.M., Marcelin J.R., Tan E.M., Rao A.K. (2018). Efficacy of Antitoxin Therapy in Treating Patients With Foodborne Botulism: A Systematic Review and Meta-Analysis of Cases, 1923–2016. Clin. Infect. Dis..

[32] Arnon S.S., Schechter R., Inglesby T.V., Henderson D.A., Bartlett J.G., Ascher M.S., Eitzen E., Fine A.D., Hauer J., Layton M. (2001). Botulinum Toxin as a Biological Weapon: Medical and Public Health Management. JAMA.

[33] McNally R.E., Morrison M.B., Bernt J.E., Stark M., Fisher J., Bo’Berry J. (1994). Effectiveness of Medical Defense Interventions Against Predicted Battlefield Level of Botulinum Toxin A..

[34] Centers for Disease Control and Prevention (CDC) (2000). Biological and Chemical Terrorism: Strategic Plan for Preparedness and Response. Recommendations of the CDC Strategic Planning Workgroup.

[35] Dembek Z.F., Smith L.A., Rusnak J.M. (2007). Botulism: Cause, Effects, Diagnosis, Clinical and Laboratory Identification, and Treatment Modalities. Disaster Med. Public Health Prep..

[36] Sobel J. (2005). Botulism. Clin. Infect. Dis..

[37] Koepke R., Sobel J., Arnon S.S. (2008). Global Occurrence of Infant Botulism, 1976–2006. Pediatrics.

[38] CDC (2019). National Botulism Surveillance Summary. https://www.cdc.gov/botulism/php/national-botulism-surveillance/2019.html.

[39] Kirk M.D., Pires S.M., Black R.E., Caipo M., Crump J.A., Devleesschauwer B., Döpfer D., Fazil A., Fischer-Walker C.L., Hald T. (2015). World Health Organization Estimates of the Global and Regional Disease Burden of 22 Foodborne Bacterial, Protozoal, and Viral Diseases, 2010: A Data Synthesis. PLoS Med..

[40] Shapiro R.L. (1998). Botulism in the United States: A Clinical and Epidemiologic Review. Ann. Intern. Med..

[41] Cox N., Hinkle R. (2002). Infant Botulism. Am. Fam. Physician.

[42] Brett M.M., Hood J., Brazier J.S., Duerden B.I., Hahné S.J.M. (2005). Soft Tissue Infections Caused by Spore-Forming Bacteria in Injecting Drug Users in the United Kingdom. Epidemiol. Infect..

[43] Centers for Disease Control and Prevention (CDC) (2006). Botulism from Home-Canned Bamboo Shoots--Nan Province, Thailand, March 2006. MMWR Morb. Mortal. Wkly. Rep..

[44] Cooper M.S., Lewis K.H., Cassel K. (1964). Antitoxins to *C. botulinum*. Proceedings of the Antitoxins to C. botulinum.

[45] Arnon S.S., Schechter R., Maslanka S.E., Jewell N.P., Hatheway C.L. (2006). Human Botulism Immune Globulin for the Treatment of Infant Botulism. N. Engl. J. Med..

[46] Richardson J.S., Parrera G.S., Astacio H., Sahota H., Anderson D.M., Hall C., Babinchak T. (2020). Safety and Clinical Outcomes of an Equine-Derived Heptavalent Botulinum Antitoxin Treatment for Confirmed or Suspected Botulism in the United States. Clin. Infect. Dis..

[47] Chalk C.H., Benstead T.J., Keezer M. (2014). Medical Treatment for Botulism. Cochrane Database Syst. Rev..

[48] Rasetti-Escargueil C., Avril A., Miethe S., Mazuet C., Derman Y., Selby K., Thullier P., Pelat T., Urbain R., Fontayne A. (2017). The European AntibotABE Framework Program and Its Update: Development of Innovative Botulinum Antibodies. Toxins.

[49] Wongtanate M., Sucharitchan N., Tantisiriwit K., Oranrigsupak P., Chuesuwan A., Toykeaw S., Suputtamongkol Y. (2007). Signs and Symptoms Predictive of Respiratory Failure in Patients with Foodborne Botulism in Thailand. Am. J. Trop. Med. Hyg..

[50] Vanella De Cuetos E.E., Fernandez R.A., Bianco M.I., Sartori O.J., Piovano M.L., Lúquez C., De Jong L.I.T. (2011). Equine Botulinum Antitoxin for the Treatment of Infant Botulism. Clin. Vaccine Immunol..

[51] Feng L., Chen X., Liu S., Zhou Z., Yang R. (2015). Two-Family Outbreak of Botulism Associated with the Consumption of Smoked Ribs in Sichuan Province, China. Int. J. Infect. Dis..

[52] Dolman C.E., Iida H. (1963). Type E Botulism: Its Epidemiology, Prevention and Specific Treatment. Can. J. Public Health.

[53] Juliao P.C., Maslanka S., Dykes J., Gaul L., Bagdure S., Granzow-Kibiger L., Salehi E., Zink D., Neligan R.P., Barton-Behravesh C. (2013). National Outbreak of Type A Foodborne Botulism Associated With a Widely Distributed Commercially Canned Hot Dog Chili Sauce. Clin. Infect. Dis..

[54] Centers for Disease Control and Prevention (CDC) (2010). Investigational Heptavalent Botulinum Antitoxin (HBAT) to Replace Licensed Botulinum Antitoxin AB and Investigational Botulinum Antitoxin E. MMWR Morb. Mortal. Wkly. Rep..

[55] Fagan R.P., Neil K.P., Sasich R., Luquez C., Asaad H., Maslanka S., Khalil W. (2011). Initial Recovery and Rebound of Type F Intestinal Colonization Botulism After Administration of Investigational Heptavalent Botulinum Antitoxin. Clin. Infect. Dis..

[56] Foster K.A. (2014). Molecular Aspects of Botulinum Neurotoxin.

[57] Rasetti-Escargueil C., Popoff M.R. (2019). Antibodies and Vaccines against Botulinum Toxins: Available Measures and Novel Approaches. Toxins.

[58] Thyagarajan B., Gopalakrishnakone P., Balali-Mood M., Llewellyn L., Singh B.R. (2015). Antidotes to Botulinum Neurotoxin. Biological Toxins and Bioterrorism.

[59] Shi D.-Y., Lu J.-S., Mao Y.-Y., Liu F.-J., Wang R., Du P., Yu S., Yu Y.-Z., Yang Z.-X. (2023). Characterization of a Novel Tetravalent Botulism Antitoxin Based on Receptor-Binding Domain of BoNTs. Appl. Microbiol. Biotechnol..

[60] Emanuel A., Qiu H., Barker D., Takla T., Gillum K., Neimuth N., Kodihalli S. (2019). Efficacy of Equine Botulism Antitoxin in Botulism Poisoning in a Guinea Pig Model. PLoS ONE.

[61] Barker D., Gillum K.T., Niemuth N.A., Kodihalli S. (2019). Therapeutic Efficacy of Equine Botulism Heptavalent Antitoxin against All Seven Botulinum Neurotoxins in Symptomatic Guinea Pigs. PLoS ONE.

[62] Kodihalli S., Emanuel A., Takla T., Hua Y., Hobbs C., LeClaire R., O’Donnell D.C. (2017). Therapeutic Efficacy of Equine Botulism Antitoxin in Rhesus Macaques. PLoS ONE.

[63] Yu P.A., Lin N.H., Mahon B.E., Sobel J., Yu Y., Mody R.K., Gu W., Clements J., Kim H.-J., Rao A.K. (2017). Safety and Improved Clinical Outcomes in Patients Treated With New Equine-Derived Heptavalent Botulinum Antitoxin. Clin. Infect. Dis..

[64] Franz D.R., Pitt L.M., Clayton M.A., Hanes M.A., Rose K.J., DasGupta B.R. (1993). Efficacy of Prophylactic and Therapeutic Administration of Antitoxin for Inhalation Botulism. Botulinum and Tetanus Neurotoxins.

[65] Van Hao N., Loan H.T., Yen L.M., Kestelyn E., Hong D.D., Thuy D.B., Nguyen N.T., Duong H.T.H., Thuy T.T.D., Nhat P.T.H. (2022). Human versus Equine Intramuscular Antitoxin, with or without Human Intrathecal Antitoxin, for the Treatment of Adults with Tetanus: A 2 × 2 Factorial Randomised Controlled Trial. Lancet Glob. Health.

[66] Aebersold P. (2012). FDA Experience with Medical Countermeasures under the Animal Rule. Adv. Prev. Med..

[67] Tacket C.O., Shandera W.X., Mann J.M., Hargrett N.T., Blake P.A. (1984). Equine Antitoxin Use and Other Factors That Predict Outcome in Type A Foodborne Botulism. Am. J. Med..

[68] Woodruff B.A., Griffin P.M., McCroskey L.M., Smart J.F., Wainwright R.B., Bryant R.G., Hutwagner L.C., Hatheway C.L. (1992). Clinical and Laboratory Comparison of Botulism from Toxin Types A, B, and E in the United States, 1975–1988. J. Infect. Dis..

[69] Black R.E., Gunn R.A. (1980). Hypersensitivity Reactions Associated with Botulinal Antitoxin. Am. J. Med..

[70] Centers for Disease Control and Prevention (CDC) (2012). Botulism from Drinking Prison-Made Illicit Alcohol—Utah 2011. MMWR Morb. Mortal. Wkly. Rep..

[71] Khouri J.M., Motter R.N., Arnon S.S. (2018). Safety and Immunogenicity of Investigational Recombinant Botulinum Vaccine, rBV A/B, in Volunteers with Pre-Existing Botulinum Toxoid Immunity. Vaccine.

[72] Robinson R.F., Nahata M.C. (2003). Management of Botulism. Ann. Pharmacother..

[73] Cheng L.W., Stanker L.H., Henderson T.D., Lou J., Marks J.D. (2009). Antibody Protection against Botulinum Neurotoxin Intoxication in Mice. Infect. Immun..

[74] Chen F., Kuziemko G.M., Amersdorfer P., Wong C., Marks J.D., Stevens R.C. (1997). Antibody Mapping to Domains of Botulinum Neurotoxin Serotype A in the Complexed and Uncomplexed Forms. Infect. Immun..

[75] Adekar S.P., Al-Saleem F.H., Elias M.D., Rybinski K.A., Simpson L.L., Dessain S.K. (2008). A Natural Human IgM Antibody That Neutralizes Botulinum Neurotoxin In Vivo. Hybridoma.

[76] Mazuet C., Dano J., Popoff M.R., Créminon C., Volland H. (2010). Characterization of Botulinum Neurotoxin Type A Neutralizing Monoclonal Antibodies and Influence of Their Half-Lives on Therapeutic Activity. PLoS ONE.

[77] Fan Y., Dong J., Lou J., Wen W., Conrad F., Geren I., Garcia-Rodriguez C., Smith T., Smith L., Ho M. (2015). Monoclonal Antibodies That Inhibit the Proteolytic Activity of Botulinum Neurotoxin Serotype/B. Toxins.

[78] Abbasova S.G., Rudenko N.V., Gorokhovatskii A.Y., Kapralova M.V., Vinogradova I.D., Vertiev Y.V., Nesmeyanov V.A., Grishin E.V. (2011). Monoclonal Antibodies to Botulinum Neurotoxins of Types A, B, E, and F. Russ. J. Bioorg. Chem..

[79] Mukherjee J., McCann C., Ofori K., Hill J., Baldwin K., Shoemaker C., Harrison P., Tzipori S. (2012). Sheep Monoclonal Antibodies Prevent Systemic Effects of Botulinum Neurotoxin A1. Toxins.

[80] Garcia-Rodriguez C., Geren I.N., Lou J., Conrad F., Forsyth C., Wen W., Chakraborti S., Zao H., Manzanarez G., Smith T.J. (2011). Neutralizing Human Monoclonal Antibodies Binding Multiple Serotypes of Botulinum Neurotoxin. Protein Eng. Des. Sel..

[81] Hatheway C.H., Snyder J.D., Seals J.E., Edell T.A., Lewis G.E. (1984). Antitoxin Levels in Botulism Patients Treated with Trivalent Equine Botulism Antitoxin to Toxin Types A, B, and E. J. Infect. Dis..

[82] Amersdorfer P., Wong C., Smith T., Chen S., Deshpande S., Sheridan R., Marks J.D. (2002). Genetic and Immunological Comparison of Anti-Botulinum Type A Antibodies from Immune and Non-Immune Human Phage Libraries. Vaccine.

[83] Wu H.-C., Yeh C.-T., Huang Y.-L., Tarn L.-J., Lung C.-C. (2001). Characterization of Neutralizing Antibodies and Identification of Neutralizing Epitope Mimics on the *Clostridium botulinum* Neurotoxin Type A. Appl. Environ. Microbiol..

[84] Lou J., Geren I., Garcia-Rodriguez C., Forsyth C.M., Wen W., Knopp K., Brown J., Smith T., Smith L.A., Marks J.D. (2010). Affinity Maturation of Human Botulinum Neurotoxin Antibodies by Light Chain Shuffling via Yeast Mating. Protein Eng. Des. Sel..

[85] Garcia-Rodriguez C., Yan S., Geren I.N., Knopp K.A., Dong J., Sun Z., Lou J., Conrad F., Wen W.-H., Farr-Jones S. (2021). A Four-Monoclonal Antibody Combination Potently Neutralizes Multiple Botulinum Neurotoxin Serotypes C and D. Toxins.

[86] Pellett S., Tepp W.H., Bradshaw M., Kalb S.R., Dykes J.K., Lin G., Nawrocki E.M., Pier C.L., Barr J.R., Maslanka S.E. (2016). Purification and Characterization of Botulinum Neurotoxin FA from a Genetically Modified Clostridium Botulinum Strain. mSphere.

[87] Nayak S.U., Griffiss J.M., McKenzie R., Fuchs E.J., Jurao R.A., An A.T., Ahene A., Tomic M., Hendrix C.W., Zenilman J.M. (2014). Safety and Pharmacokinetics of XOMA 3AB, a Novel Mixture of Three Monoclonal Antibodies against Botulinum Toxin A. Antimicrob. Agents Chemother..

[88] Search for: Other Terms: Xoma 3ab|Card Expert Search|ClinicalTrials.Gov. https://clinicaltrials.gov/expert-search?term=xoma%203ab.

[89] Lam K., Tremblay J.M., Perry K., Ichtchenko K., Shoemaker C.B., Jin R. (2022). Probing the Structure and Function of the Protease Domain of Botulinum Neurotoxins Using Single-Domain Antibodies. PLoS Pathog..

[90] Tremblay J.M., Vazquez-Cintron E., Lam K.-H., Mukherjee J., Bedenice D., Ondeck C.A., Conroy M.T., Bodt S.M.L., Winner B.M., Webb R.P. (2020). Camelid VHH Antibodies That Neutralize Botulinum Neurotoxin Serotype E Intoxication or Protease Function. Toxins.

[91] Harmsen M.M., Cornelissen J.C., Van Der Wal F.J., Bergervoet J.H.W., Koene M. (2023). Single-Domain Antibody Multimers for Detection of Botulinum Neurotoxin Serotypes C, D, and Their Mosaics in Endopep-MS. Toxins.

[92] Godakova S.A., Noskov A.N., Vinogradova I.D., Ugriumova G.A., Solovyev A.I., Esmagambetov I.B., Tukhvatulin A.I., Logunov D.Y., Naroditsky B.S., Shcheblyakov D.V. (2019). Camelid VHHs Fused to Human Fc Fragments Provide Long Term Protection Against Botulinum Neurotoxin A in Mice. Toxins.

[93] Barash J.R., Arnon S.S. (2014). A Novel Strain of Clostridium Botulinum That Produces Type B and Type H Botulinum Toxins. J. Infect. Dis..

[94] Maslanka S.E., Lúquez C., Dykes J.K., Tepp W.H., Pier C.L., Pellett S., Raphael B.H., Kalb S.R., Barr J.R., Rao A. (2016). A Novel Botulinum Neurotoxin, Previously Reported as Serotype H, Has a Hybrid-Like Structure With Regions of Similarity to the Structures of Serotypes A and F and Is Neutralized With Serotype A Antitoxin. J. Infect. Dis..

[95] Zhang S., Masuyer G., Zhang J., Shen Y., Lundin D., Henriksson L., Miyashita S.-I., Martínez-Carranza M., Dong M., Stenmark P. (2017). Identification and Characterization of a Novel Botulinum Neurotoxin. Nat. Commun..

[96] Kitasato S. (1889). Ueber Den Tetanusbacillus. Z. Hyg..

[97] Rossetto O., Scorzeto M., Megighian A., Montecucco C. (2013). Tetanus Neurotoxin. Toxicon.

[98] Schiavo G., Papini E., Genna G., Montecucco C. (1990). An Intact Interchain Disulfide Bond Is Required for the Neurotoxicity of Tetanus Toxin. Infect. Immun..

[99] Cheng K., Lu J., Guo J., Wang R., Chen L., Wang X., Jiang Y., Li Y., Xu C., Kang Q. (2024). Characterization of Neutralizing Chimeric Heavy-Chain Antibodies against Tetanus Toxin. Hum. Vaccines Immunother..

[100] Tetanus. https://www.ecdc.europa.eu/en/tetanus.

[101] Li J., Liu Z., Yu C., Tan K., Gui S., Zhang S., Shen Y. (2023). Global Epidemiology and Burden of Tetanus from 1990 to 2019: A Systematic Analysis for the Global Burden of Disease Study 2019. Int. J. Infect. Dis..

[102] Tetanus. https://www.who.int/health-topics/tetanus.

[103] Tetanus Vaccines: WHO Position Paper—February 2017. https://www.who.int/publications/i/item/WHO-WER9206.

[104] Orenstein W. (2023). Plotkin’s Vaccines, e-Book.

[105] Behring H., Kitasato H. (1890). Ueber das Zustandekommen der Diphtherie-Immunität und der Tetanus-Immunität bei Thieren. Dtsch. Med. Wochenschr..

[106] Nocard E. (1895). Sur La Sérothérapie Du Tétanos. Essais de Traitement Préventif. Bull. Acad. Med..

[107] Nocard E. (1897). Sur La Sérothérapie Du Tétanos. Receuil Méd. Vét..

[108] Hewlett R.T. (1895). Tetanus Antitoxin; Its Preparation and Properties. BMJ.

[109] Struthers J.W. (1908). The Treatment of Tetanus. Edinb. Med. J..

[110] Aubert N., Brachet-Botineau M., de Olivera Preto G.E., Benz-de Bretagne I., Watier H., Brachet G. (2020). History, Extensive Characterization and Challenge of Anti-Tetanus Serum from World War I: Exciting Remnants and Deceived Hopes: Centenarian IgGs Lost Their Neutralization Capacity. Immunol. Res..

[111] Wever P.C., Van Bergen L. (2012). Prevention of Tetanus during the First World War. Med. Humanit..

[112] Buer A.W. (1946). A Method for Production of High-Titre Tetanus Serum. Acta Pathol. Microbiol. Scand..

[113] Ramon G. (1923). Médecine Expérimentale. Sur Le Pouvoir Floculant et Sur Les Propriétés Immunisantes d’une Toxine Diphtérique Rendue Anatoxique (Anatoxine). CR Acad. Sci..

[114] Ramon G. (1925). Sur l’augmentation Anormale de l’antitoxine Chez Les Chevaux Producteurs de Sérum Antidiphtérique. Bull. Soc. Centr. Med. Vet..

[115] Pope C.G. (1963). Development of Knowledge of Antitoxins. Br. Med. Bull..

[116] Lang J., Kamga-Fotso L., Peyrieux J.C., Blondeau C., Lutsch C., Forrat R. (2000). Safety and Immunogenicity of a New Equine Tetanus Immunoglobulin Associated with Tetanus-Diphtheria Vaccine. Am. J. Trop. Med. Hyg..

[117] McCracken G.H., Dowell D.L., Marshall F.N. (1971). Double-Blind Trial of Equine Antitoxin and Human Immune Globulin in Tetanus Neonatorum. Lancet.

[118] Rubbo S.D., Suri J.C. (1962). Passive Immunization Against Tetanus with Human Immune Globulin. BMJ.

[119] Yu R., Ji C., Xu J., Wang D., Fang T., Jing Y., Kwang-Fu Shen C., Chen W. (2018). The Immunogenicity of the C Fragment of Tetanus Neurotoxin in Production of Tetanus Antitoxin. BioMed Res. Int..

[120] Joseph M., Woldeamanuel Y., Medhin G., Manyazewal T., Fekadu A., Makonnen E. (2023). Safety of Equine Tetanus Antitoxin for Prophylactic Use in Ethiopia: A Retrospective Multi-Center Study. Trop. Med. Health.

[121] Bennett J.E., Dolin R., Blaser M.J. (2020). Mandell, Douglas, and Bennett’s Principles and Practice of Infectious Diseases.

[122] Nation N.S., Pierce N.F., Adler S.J., Chinnock R.F., Wehrle P.F. (1963). Tetanus; the Use of Human Hyperimmune Globulin in Treatment. Calif. Med..

[123] Blake P.A., Feldman R.A., Buchanan T.M., Brooks G.F., Bennett J.V. (1976). Serologic Therapy of Tetanus in the United States, 1965–1971. JAMA.

[124] Afshar M., Raju M., Ansell D., Bleck T.P. (2011). Narrative Review: Tetanus—A Health Threat After Natural Disasters in Developing Countries. Ann. Intern. Med..

[125] Cook T.M., Protheroe R.T., Handel J.M. (2001). Tetanus: A Review of the Literature. Br. J. Anaesth..

[126] Farrar J.J. (2000). Neurological Aspects of Tropical Disease: Tetanus. J. Neurol. Neurosurg. Psychiatry.

[127] Thwaites C.L., Yen L.M., Jameson J.L., Fauci A.S., Kasper D.L., Hauser S.L., Longo D.L., Loscalzo J. (2018). Tetanus. Harrison’s Principles of Internal Medicine.

[128] Abrutyn E., Berlin J.A. (1991). Intrathecal Therapy in Tetanus. A Meta-Analysis. JAMA.

[129] Begue R.E., Lindo-Soriano I. (1991). Failure of Intrathecal Tetanus Antitoxin in the Treatment of Tetanus Neonatorum. J. Infect. Dis..

[130] Kabura L., Ilibagiza D., Menten J., Van Den Ende J. (2006). Intrathecal vs. Intramuscular Administration of Human Antitetanus Immunoglobulin or Equine Tetanus Antitoxin in the Treatment of Tetanus: A Meta-analysis. Trop. Med. Int. Health.

[131] Agarwal M., Thomas K., Peter J.V., Jeyaseelan L., Cherian A.M. (1998). A Randomized Double-Blind Sham-Controlled Study of Intrathecal Human Anti-Tetanus Immunoglobulin in the Management of Tetanus. Natl. Med. J. India.

[132] Geeta M.G., Krishnakumar P., Mathews L. (2007). Intrathecal Tetanus Immunoglobulins in the Management of Tetanus. Indian J. Pediatr..

[133] Miranda-Filho D.d.B., Ximenes R.A.d.A., Barone A.A., Vaz V.L., Vieira A.G., Albuquerque V.M.G. (2004). Randomised Controlled Trial of Tetanus Treatment with Antitetanus Immunoglobulin by the Intrathecal or Intramuscular Route. BMJ.

[134] Rossotti M.A., González-Techera A., Guarnaschelli J., Yim L., Camacho X., Fernández M., Cabral P., Leizagoyen C., Chabalgoity J.A., González-Sapienza G. (2015). Increasing the Potency of Neutralizing Single-Domain Antibodies by Functionalization with a CD11b/CD18 Binding Domain. MAbs.

[135] de Smit H., Ackerschott B., Tierney R., Stickings P., Harmsen M.M. (2021). A Novel Single-Domain Antibody Multimer That Potently Neutralizes Tetanus Neurotoxin. Vaccine X.

[136] Hadfield T.L., McEvoy P., Polotsky Y., Tzinserling V.A., Yakovlev A.A. (2000). The Pathology of Diphtheria. J. Infect. Dis..

[137] Collier R.J. (1975). Diphtheria Toxin: Mode of Action and Structure. Bacteriol. Rev..

[138] Ryan K.J., Ray C.G., Sherris J.C. (2004). Sherris Medical Microbiology: An Introduction to Infectious Diseases.

[139] Holmes R.K. (2000). Biology and Molecular Epidemiology of Diphtheria Toxin and the *Tox* Gene. J. Infect. Dis..

[140] Pappenheimer A.M. (1984). The Diphtheria Bacillus and Its Toxin: A Model System. J. Hyg..

[141] Popovic T., Mazurova I.K., Efstratiou A., Vuopio-Varkila J., Reeves M.W., De Zoysa A., Glushkevich T., Grimont P. (2000). Molecular Epidemiology of Diphtheria. J. Infect. Dis..

[142] Wagner K.S., White J.M., Lucenko I., Mercer D., Crowcroft N.S., Neal S., Efstratiou A., on behalf of the Diphtheria Surveillance Network (2012). Diphtheria in the Postepidemic Period, Europe, 2000–2009. Emerg. Infect. Dis..

[143] Clarke K.E.N. Review of the Epidemiology of Diphtheria—2000–2016. https://cdn.who.int/media/docs/default-source/immunization/sage/2017/sage-meeting-of-april-2017/background-docs/session-diphtheria/1.-review-of-the-epidemiology-of-diphtheria---2000-2016-pdf-829kb.pdf?sfvrsn=9ba4f061_3.

[144] Diphtheria—Number of Reported Cases. https://www.who.int/data/gho/data/indicators/indicator-details/GHO/diphtheria---number-of-reported-cases.

[145] Kolybo D.V., Labyntsev A.A., Romaniuk S.I., Kaberniuk A.A., Oliinyk O.M., Korotkevich N.V., Komisarenko S.V. (2013). Immunobiology of Diphtheria. Recent Approaches for the Prevention, Diagnosis, and Treatment of Disease. Biotechnol. Acta.

[146] Kneen R., Giao P.N., Solomon T., Van T.T.M., Hoa N.T.T., Long T.B., Wain J., Day N.P.J., Hien T.T., Parry C.M. (1998). Penicillin vs. Erythromycin in the Treatment of Diphtheria. Clin. Infect. Dis..

[147] Clinical Management of Diphtheria: Guideline, 2 February 2024. https://www.who.int/publications/i/item/WHO-DIPH-Clinical-2024.1.

[148] Diphtheria-Guinea. https://www.who.int/emergencies/disease-outbreak-news/item/2023-DON492.

[149] Linton D.S. (2005). Emil von Behring: Infectious Disease, Immunology, Serum Therapy.

[150] Schwartz R.S. (2004). Paul Ehrlich’s Magic Bullets. N. Engl. J. Med..

[151] Wagner K.S., Stickings P., White J.M., Neal S., Crowcroft N.S., Sesardic D., Efstratiou A. (2009). A Review of the International Issues Surrounding the Availability and Demand for Diphtheria Antitoxin for Therapeutic Use. Vaccine.

[152] Hassall L., Rigsby P., Stickings P. (2023). Collaborative Study for the Calibration of a Replacement International Standard for Diphtheria Antitoxin Equine. Biologicals.

[153] Wenzel E.V., Bosnak M., Tierney R., Schubert M., Brown J., Dübel S., Efstratiou A., Sesardic D., Stickings P., Hust M. (2020). Human Antibodies Neutralizing Diphtheria Toxin in Vitro and in Vivo. Sci. Rep..

[154] Kniker W.T., Cochrane C.G. (1968). The Localization of Circulating Immune Complexes in Experimental Serum Sickness. J. Exp. Med..

[155] Bissumbhar B., Rakhmanova A.G., Berbers G.A.M., Iakolev A., Nosikova E., Melnick O., Ovtcharenko E., Rümke H.C., Ruitenberg E.J. (2004). Evaluation of Diphtheria Convalescent Patients to Serve as Donors for the Production of Anti-Diphtheria Immunoglobulin Preparations. Vaccine.

[156] Provan D., Chapel H.M., Sewell W.A.C., O’Shaughnessy D., on behalf of the UK Immunoglobulin Expert Working Group (2008). Prescribing Intravenous Immunoglobulin: Summary of Department of Health Guidelines. BMJ.

[157] Kakita M., Takahashi T., Komiya T., Iba Y., Tsuji T., Kurosawa Y., Takahashi M. (2006). Isolation of a Human Monoclonal Antibody with Strong Neutralizing Activity against Diphtheria Toxin. Infect. Immun..

[158] Sevigny L.M., Booth B.J., Rowley K.J., Leav B.A., Cheslock P.S., Garrity K.A., Sloan S.E., Thomas W., Babcock G.J., Wang Y. (2013). Identification of a Human Monoclonal Antibody To Replace Equine Diphtheria Antitoxin for Treatment of Diphtheria Intoxication. Infect. Immun..

[159] Smith H.L., Cheslock P., Leney M., Barton B., Molrine D.C. (2016). Potency of a Human Monoclonal Antibody to Diphtheria Toxin Relative to Equine Diphtheria Anti-Toxin in a Guinea Pig Intoxication Model. Virulence.

[160] Sullivan-Bólyai J.Z., Allen L.B., Cannon R., Cohane K.P., Dunzo E., Goldwater R., StCyr K., Wang Y., Klempner M.S. (2024). A Phase 1 Study in Healthy Subjects to Evaluate the Safety and Pharmacokinetics of a Human Monoclonal Antibody (S315) Against Diphtheria Toxin. J. Infect. Dis..

[161] Khalili E., Lakzaei M., Aminian M. (2024). Neutralizing Anti-Diphtheria Toxin scFv Produced by Phage Display. Biotechnol. Lett..

[162] Shaker G. (2010). Evaluation of Antidiphtheria Toxin Nanobodies. Nanotechnol. Sci. Appl..

[163] Zhu J., Declercq J., Roucourt B., Ghassabeh G.H., Meulemans S., Kinne J., David G., Vermorken A.J.M., Van De Ven W.J.M., Lindberg I. (2012). Generation and Characterization of Non-Competitive Furin-Inhibiting Nanobodies. Biochem. J..

[164] Rubio-Casillas A., Rodriguez-Quintero C.M., Redwan E.M., Gupta M.N., Uversky V.N., Raszek M. (2024). Do Vaccines Increase or Decrease Susceptibility to Diseases Other than Those They Protect Against?. Vaccine.

[165] Smith L.A. (2009). Botulism and Vaccines for Its Prevention. Vaccine.

[166] Torii Y., Sugimoto N., Kohda T., Kozaki S., Morokuma K., Horikawa Y., Ginnaga A., Yamamoto A., Takahashi M. (2017). Clinical Study of New Tetravalent (Type A, B, E, and F) Botulinum Toxoid Vaccine Derived from M Toxin in Japan. Jpn. J. Infect. Dis..

[167] Enria L., Dwyer H., Marchant M., Beckmann N., Schmidt-Sane M., Conteh A., Mansaray A., N’Jai A. (2024). Political Dimensions of Misinformation, Trust, and Vaccine Confidence in a Digital Age. BMJ.

[168] Confidence in Childhood Vaccines Declines Across Europe and Central Asia—New UNICEF Report. https://www.unicef.org/eca/press-releases/confidence-childhood-vaccines-declines-across-europe-and-central-asia-new-unicef.

